# Exploring the Role of *CCNF* Variants in Italian ALS Patients

**DOI:** 10.3390/genes15121566

**Published:** 2024-12-03

**Authors:** Giulia Bisogni, Amelia Conte, Umberto Costantino, Serena Lattante, Daniela Bernardo, Gabriele Lucioli, Agata Katia Patanella, Paola Cimbolli, Elda Del Giudice, Federica Vettor, Giuseppe Marangi, Paolo Niccolò Doronzio, Marcella Zollino, Mario Sabatelli

**Affiliations:** 1 Centro Clinico NeMO Adulti, Fondazione Serena Onlus-Fondazione Policlinico Universitario Agostino Gemelli IRCCS, 00168 Rome, Italy; amelia.conte@centrocliniconemo.it (A.C.); daniela.bernardo@centrocliniconemo.it (D.B.); gabriele.lucioli@centrocliniconemo.it (G.L.); katia.patanella@centrocliniconemo.it (A.K.P.); paola.cimbolli@centrocliniconemo.it (P.C.); mario.sabatelli@unicatt.it (M.S.); 2 Neurology Unit, Fondazione IRCCS-Casa Sollievo della Sofferenza, San Giovanni Rotondo, 71013 Foggia, Italy; umbertocostantino94@gmail.com; 3 Department of Experimental Medicine, Università del Salento, 73100 Lecce, Italy; serena.lattante@unisalento.it; 4 Research & Innovation (R&I Genetics) Srl, 35127 Padova, Italy; edelgiudice@rigenetics.com (E.D.G.); fvettor@rigenetics.com (F.V.); 5 Section of Genomic Medicine, Department of Life Sciences and Public Health, Università Cattolica del Sacro Cuore, 00168 Rome, Italy; giuseppe.marangi@unicatt.it (G.M.); paoloniccolo.doronzio@unicatt.it (P.N.D.); marcella.zollino@unicatt.it (M.Z.); 6 Unit of Medical Genetics, Department of Laboratory and Infectious Disease Sciences, Fondazione Policlinico Universitario A. Gemelli IRCCS, 00168 Rome, Italy; 7 Institute of Neurology, Università Cattolica del Sacro Cuore, 00168 Rome, Italy

**Keywords:** amyotrophic lateral sclerosis, motor neuron disease, FTD, Cyclin F

## Abstract

**Objectives:** Variants in Cyclin F (*CCNF*) have been associated to amyotrophic lateral sclerosis (ALS) and/or frontotemporal dementia (FTD) in a group of cases. The objectives of this study were to determine the contribution of *CCNF* in a large cohort of Italian ALS patients, to look for genotype-phenotype correlation of the mutations and to evaluate the *CCNF*-associated clinical features. **Methods:** We applied next-generation sequencing technologies on 971 unrelated Italian ALS patients and we filtered results to look for variants in *CCNF* gene. **Results:** We identified 13 rare missense variants in 16 index cases (2 familial and 14 sporadic), with a cumulative mutational frequency of 1.6%. The most prevalent variant was p.Phe197Leu, found in three patients. The clinical presentation was heterogeneous, with a classic phenotype in eight patients, upper motor neuron dominant (UMN-D) phenotype in four patients, and flail arm in four patients. Clinical evaluation for cognitive impairment was performed in 13 patients using the Edinburgh Cognitive and Behavioural ALS Screen (ECAS) test, demonstrating that almost half of the patients (*n* = 6) had variable degrees of frontal dysfunction. **Discussion:** In our cohort, we observed *CCNF* variants in 1.6% of patients (16/971), a percentage similar to that found in other series. Clinical presentation is heterogeneous, but *CCNF* variants are significantly associated to cognitive impairment. **Conclusions:** Our study expands the *CCNF* genetic variant spectrum in a large cohort of Italian ALS patients. Further studies are needed to assess genotype-phenotype associations of *CCNF* variants and to specify the role of each variant, which are quite common, especially in sALS patients.

## 1. Introduction

Amyotrophic lateral sclerosis (ALS) is a fatal neurodegenerative disease that causes progressive degeneration of upper motor neurons (UMNs) in the cerebral cortex and lower motor neurons (LMNs) in the brainstem and the spinal cord. In the majority of patients (90%), the disease is apparently sporadic (sALS), while familial ALS represents about 10% of cases (fALS). Clinically, the primary symptoms of ALS are distinguished by involvement of different sets of motor neurons or different regions of the body. Importantly, ALS shows clinical overlap with other neurological disorders. Cognitive and/or behavioural disorders may involve up to 50% of patients during the course of the disease and a concomitant behavioural variant of frontotemporal dementia (bv-FTD) has been observed in 13% of ALS patients [1,2]. Genetically, more than 100 genes are involved in the pathogenesis of ALS, although four genes (*SOD1*, *C9orf72*, *FUS* and *TARDBP*) account for up to 60% of fALS and 15% of sALS, acting as “major ALS genes” [3].

In 2016, a heterozygous missense mutation in the *CCNF* gene was identified by coupling whole-genome linkage analysis with whole-exome sequencing as the causative gene of ALS/FTD in a large Australian family of British descent. Subsequent extensive screening of several cohorts with different geographic origins revealed additional, potentially pathogenic variants in *CCNF*. Furthermore, analysis in a replication cohort demonstrated a significant enrichment of novel and rare protein-altering *CCNF* variants in sALS patients [4]. *CCNF* encodes a member of the cyclin family, the 786 amino-acid cyclin F protein, first identified in 1994 [5]. Cyclin F, a component of the Skp1-Cul1-F-box E3 ubiquitin ligase complex SCF^cyclin F^ [6], is a member of the F-box protein family and uses the 40 amino acid F-box domain to promote the ubiquitylation of target substrates [7], thus playing a key role in ubiquitin-proteasome mediated protein degradation. Dysfunction of cyclin F has been implicated in various forms of cancer and may be involved in neurodegeneration [6].

Williams and colleagues [4] demonstrated that expression of mutant *CCNF* in neuronal cells caused abnormal ubiquitination and accumulation of ubiquitinated proteins, including TDP-43 and a SCF^Cyclin F^ substrate.

Currently, the pathogenetic mechanisms underlying neurodegeneration remain poorly characterized for cyclin F-associated ALS/FTD. Recently, a study by Ragagnin et al. [8] provided novel insights into these mechanisms, demonstrating that ALS/FTD-associated variant cyclin F^S621G^ perturbs both secretory protein trafficking and the endoplasmic reticulum (ER)–Golgi apparatus homeostasis, inducing ER stress, ERAD (endoplasmic reticulum associated degradation), and Golgi fragmentation.

In this study, we looked for *CCNF* variants in a large cohort of Italian ALS patients to further understand the contribution of these variants to ALS pathogenesis and to describe associated clinical features.

## 2. Patients and Methods

A cohort of 971 unrelated Italian ALS patients admitted to NEMO Clinical Center, an ALS Referral Center located in Rome, was enrolled in this study from 2018 to 2024. The study conformed with the World Medical Association Declaration of Helsinki and was approved by the Ethics Committee of our Institution (prot. A.133/C.E./2013). All recruited patients signed an informed consent. ALS diagnosis was made according to El Escorial criteria [9] by expert neurologists.

After accurate investigation of familiarity for the disease, two patients were classified as familial cases and fourteen as sporadic cases. For each family, only one index case was considered. Patients were clinically categorized into four different phenotypes: classic, upper motor neuron dominant (UMN-D), flail arm, and pure LMN [10]. The Kaplan–Meier method was used for survival analysis, and the survival time was calculated from disease onset to last follow-up, death, or tracheostomy.

The presence of cognitive impairment was deeply investigated via administration of the Edinburgh Cognitive and Behavioural Amyotrophic Lateral Sclerosis Screen (ECAS) Italian version [11] to a group of patients. The coexistence of cognitive and/or behavioural involvement was assessed in accordance with the revised criteria for the diagnosis of frontotemporal dysfunction in ALS [12]: normal cognition, ALS with cognitive impairment (ALSci), ALS with behavioural impairment (ALSbi), ALS with combined cognitive and behavioural impairment (ALScbi), and ALS with frontotemporal dementia (ALS-FTD).

### Genetic Analysis

After written informed consent had been obtained, blood samples were taken from patients. Genomic DNA were extracted using standard protocols and tested at Research and Innovation (R&I Genetics) srl, Padua, Italy. A panel of 141 genes associated to motor neuron diseases was analyzed using next-generation sequencing technologies. The Sure Select all exon V.6 kit (Agilent Technologies, Inc., Santa Clara, CA, USA) was used to prepare the DNA library, and the HiSeq2500 Sequencer (Illumina, San Diego, California) was used for the sequencing. Genetic variants in *CCNF* (*NM_001761*) were extracted from all data and confirmed by Sanger sequencing. Variants were categorized using the Franklin variant interpretation platform (https://franklin.genoox.com, accessed on 20 October 2024), according to the ACMG criteria. The presence of the pathogenic hexanucleotide repeat expansion in the *C9orf72* gene was investigated in all patients by repeat-primed PCR.

## 3. Results

We identified 13 rare variants in 16 index cases (2 fALS and 14 sALS), with a cumulative mutational frequency of 1.6%. All the identified variants were in a heterozygous state. The most prevalent variant was p.Phe197Leu, found in three patients (Table 1).

Based on ACMG recommendations, no variants were classified as certainly pathogenic, but they were classified as “variants of uncertain significance” (VUS). All the variants we identified in our cohort were extremely rare in control populations, and the allelic frequency ranged from 0 to 0.000007967 reported on gnomAD. Only one variant had been previously identified in ALS patients (Table 1). Unfortunately, we were unable to perform familial segregation analysis. One patient carried also the p.Gln380Glu variant in *NEK1*, and another carried a concomitant *C9orf72* expansion.

**Figure 1:**
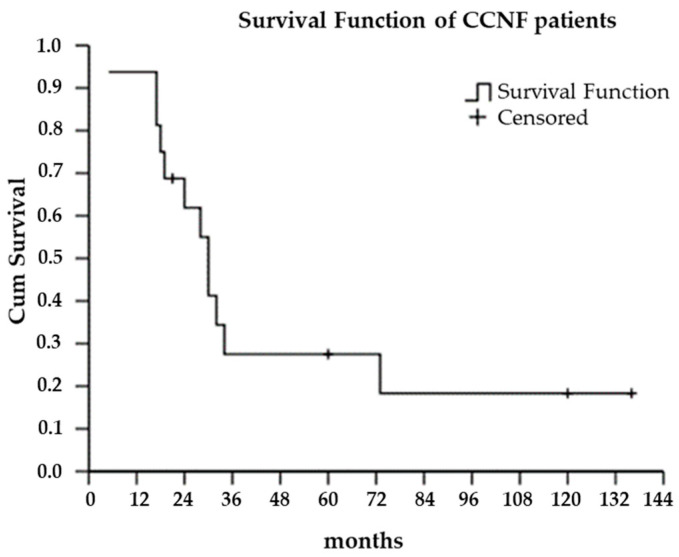
Kaplan–Meier curve of survival in *CCNF* patients.

The clinical characteristics of our patients carrying *CCNF* variants are shown in Table 1. Clinical presentation was heterogeneous, with eight patients presenting with a classic phenotype, four patients showing the UMN-D phenotype, and four patients with flail arm. The majority of patients had a spinal onset (68.7%, five upper limb and six lower limb), while the remaining five patients (31.3%) had a bulbar onset. The male/female ratio was 13/3. The mean age at onset was 66 ± 9.7 (range 48–76) years, and the median survival time was 30 months (Figure 1).

Clinical evaluation for cognitive impairment using the Edinburgh Cognitive and Behavioural ALS Screen (ECAS) test was performed for thirteen patients, demonstrating that six patients had cognitive and/or behavioural impairment: three had overt FTD, two had ALSci, and one patient had ALSbi. It should be noted that one patient (Patient 1684) with FTD had a concomitant *C9orf72* repeat expansion and a family history of cognitive impairment.

In our cohort, the most frequent variant was p.Phe197Leu, found in three patients (two males and one female): one fALS (possible) and two sALS. All three patients had a spinal onset and the following phenotypes: one classic phenotype, one UMN-D phenotype, and one flail arm phenotype. The mean age at onset of patients carrying this variant was 67.7 ± 5.1 (range 62–72). Cognitive impairment was not detected in these patients.

The second most frequent variant, p.Asn87Lys, was identified in two male sALS patients, both with spinal onset and flail arm phenotype. The mean age at onset of patients carrying the p.Asn87Lys variant was 58 ± 12.7 years.

The clinical characteristics of the patients carrying the remaining 11 variants, all found in single patients, are detailed in Table 1.

## 4. Discussion

In the present study, we examined the clinical phenotype and genetic spectrum of *CCNF* variants through a large Italian ALS cohort. To our knowledge, this is the largest existing investigation of the *CCNF* gene in Italian ALS patients.

In our cohort of 971 patients, a total of 16 ALS patients were carriers of 13 different missense variants, with a cumulative mutational frequency of 1.6%, comparable to that previously reported in other populations [4,13,14,15,16,17,18]. None of these variants is classified as clearly pathogenic since they are not described in other ALS patients, except for p.Ser509Pro [4], and bioinformatic tools gave mostly benign predictions. However, they are classified as “variants of uncertain significance” since they are extremely rare in the control population.

Furthermore, *CCNF* variants can have an impact on protein function, as suggested by their location. Cyclin F protein contains three functional modules: the F-box domain, which is a catalytic module; the two cyclin domains, forming the substrate recruitment module, and the C-terminal regulatory module containing a nuclear localization signal and a PEST sequence (enriched in proline, glutamic acid, serine and threonine). In our cohort, eight variants were located in functional domains: four were located in the cyclin domain (p.Asp307Gly and p.Ala400Thr in the N-terminal and p.Thr478Ile and p.Ser509Pro in the C-terminal portion) and four were in the PEST domain (p.Pro565Thr, p.Ser566Leu, p.Ala658Val and p.Ser732Gly).

Clinical manifestations were heterogeneous among our patients, as disease duration varied from less than one year to 10 years and the age of onset ranged from 48 to 76 years. The site of onset was spinal in the majority of patients (68.7%). Two patients were classified as fALS and fourteen as sALS. Among fALS patients, one patient (Patient 1684) had a concomitant *C9orf72* repeat expansion.

Clinical evaluation for cognitive impairment using the Edinburgh Cognitive and Behavioural ALS Screen (ECAS) test demonstrated that 6/13 patients had cognitive and/or behavioural impairments, thus confirming, as previously reported [13], that *CCNF* variants are significantly associated to cognitive impairment.

In our cohort, the most frequent variant was p.Phe197Leu, accounting for 18.7% of mutated cases (3/16). One patient carried a concomitant p.Gln380Glu variant in *NEK1*. Interestingly, cognitive impairment was not detected in patients carrying the p.Phe197Leu variant. The second most frequent variant, p.Asn87Lys, was identified in two patients with flail arm phenotype.

Our study has some limitations. First, the pathogenicity of the identified *CCNF* variants is not clear, but they are extremely rare and most of them are located in functional domains. Second, we could not perform familial segregation analysis in order to determine if these variants segregate with disease. Nevertheless, our findings broaden the *CCNF* genetic variant spectrum, and confirm their association with cognitive impairment. Variants in the *CCNF* gene, as well as in other ALS-associated “minor” genes, may not be sufficient by themselves to cause the disease, but they can contribute to ALS pathogenesis, acting as a predisposing factor and combining with other genetic and/or environmental factors. Further studies are needed to assess the pathogenetic role of *CCNF* variants in ALS.

## Figures and Tables

**Table 1:** *CCNF* variants and clinical characteristics of our cohort.

Patient ID	Nucleotidic Change	Aminoacidic Change	Reference	gnomAD Exomes Global Allele Frequency	Additional Variants	Disease Duration (Months)	Outcome	Phenotype	SALS/ FALS	Cognitive/ Behavioural Impairment
833	c.261C > A	p.Asn87Lys	-	0.0000206		136	Alive	Flail arm	SALS	No
2496	c.261C > A	p.Asn87Lys	-	0.0000206		21	Lost at follow-up	Flail arm	SALS	N/A
2269	c.467C > T	p.Pro156Leu	-	0.00000891		17	Deceased	Classic	SALS	Yes (ALSci)
2354	c.591C > A	p.Phe197Leu	-	0.0000193		60	Alive	Flail arm	FALS (possible)	No
1897	c.591C > A	p.Phe197Leu	-	0.0000193	*NEK1* p.Gln380Glu	28	Deceased	Classic	SALS	No
anv-1842	c.591C > A	p.Phe197Leu	-	0.0000193		120	Alive	UMN-D	SALS	No
2097	c.656T > C	p.Leu219Pro	-	0.0000165		17	Deceased	Classic	SALS	N/A
1713	c.697G > C	p.Asp233His	-	0.0000317		73	Deceased	UMN-D	SALS	Yes (ALS-FTD)
2532	c.920A > G	p.Asp307Gly	-	0		19	Deceased	Flail arm	SALS	No
deg-2712	c.1198G > A	p.Ala400Thr	-	0.00000684		30	Deceased	UMN-D	SALS	Yes (ALSbi)
2233	c.1433C > T	p.Thr478Ile	-	0.000000712		34	Deceased	UMN-D	SALS	No
2032	c.1525T > C	p.Ser509Pro	4	0.00000616		24	Deceased	Classic	SALS	No
1684	c.1693C > A	p.Pro565Thr	-	0.0000445	*C9orf72* expansion	30	Deceased	Classic	FALS (definite)	Yes (ALS-FTD)
1901	c.1697C > T	p.Ser566Leu	-	0.0000164		18	Deceased	Classic	SALS	Yes (ALSci)
1795	c.1973C > T	p.Ala658Val	-	0.0000158		5	Tracheostomy (deceased after 29 m)	Classic	SALS	N/A
1741	c.2194A > G	p.Ser732Gly	-	0.0000041		32	Deceased	Classic	SALS	Yes (ALS-FTD)

Abbreviations: ALS, amyotrophic lateral sclerosis; ALSbi, ALS with behavioural impairment; ALSci, ALS with cognitive impairment; FALS, familial ALS; FTD, frontotemporal dementia; m, months; N/A, not available; SALS, sporadic ALS.

## Data Availability

Dataset available from the corresponding author upon reasonable request.
